# Baseline knowledge on risk factors, symptoms and intended behavior of women and men towards screening and treatment of cervical cancer in rural Uganda: a cross-sectional study

**DOI:** 10.1186/s12885-024-12223-8

**Published:** 2024-04-11

**Authors:** Carolyn Nakisige, Marlieke de Fouw, Miriam Nakalembe, Jackson Orem, Dan Atukonyera, Mwalimu Musheshe, Jaap Koot, Janine de Zeeuw, Jogchum Beltman, Jelle Stekelenburg

**Affiliations:** 1 Department of Gynaecologic-Oncology, Uganda Cancer Institute, Kampala, Uganda; 2 Department of Gynaecology, Leiden University Medical Centre, Leiden, The Netherlands; 3 Makerere University College of Medicine, Kampala, Uganda; 4 Uganda Rural Development Training Program, Kagadi, Uganda; 5 Department of Health Sciences, Global Health Unit, University Medical Center Groningen, University of Groningen, Groningen, The Netherlands; 6 Department of Obstetrics and Gynecology, Medical Centre Leeuwarden, Leeuwarden, The Netherlands

**Keywords:** Risk factors, Symptoms, Intended behavior, Cervical cancer screening

## Abstract

**Background:**

Knowledge of risk factors and symptoms of cervical cancer has been found to promote uptake of screening of cervical cancer. Most interventions targeted women without much involvement of men (husbands/decision makers) who are often decision makers in many low- and middle-income countries. This study aimed at assessing baseline knowledge and intended behavior of both women and men to enable design specific targeted messages to increase uptake of cervical cancer screening and promote early detection of women with symptoms.

**Methods:**

This cross-sectional study was conducted in two districts in Western Uganda using the modified African Women Awareness of CANcer (AWACAN) questionnaire. Women aged 30—49 years and their husbands/decision makers were interviewed. Knowledge on risk factors and symptoms, intended behavior and barriers towards participation in cervical cancer screening and treatment were assessed. Descriptive and logistic regression analyses were done to establish the association between knowledge levels and other factors comparing women to men.

**Results:**

A total of 724 women and 692 men were enrolled. Of these, 71.0% women and 67.2% men had ever heard of cervical cancer and 8.8% women had ever been screened. Knowledge of risk factors and symptoms of cervical cancer was high and similar for both women and men.

Lack of decision making by women was associated with low knowledge of risk factors (X^2^ = 14.542; *p* = 0.01), low education (X^2^ = 36.05, *p* < 0.01) and older age (X^2^ = 17.33, *p* < 0.01). Men had better help seeking behavior than women (X^2^ = 64.96, *p* < 0.01, OR = 0.39, 95% CI: 0.31—0.50) and were more confident and skilled in recognising a sign or symptom of cervical cancer (X^2^ = 27.28, *p* < 0.01, OR = 0.52, CI (0.40—0.67).

**Conclusion:**

The baseline knowledge for cervical cancer was high in majority of participants and similar in both women and men. Their intended behavior towards screening was also positive but screening uptake was very low. This study suggests developing messages on multiple interventions to promote screening behavior in addition to education, consisting of male involvement, women empowerment and making services available, accessible and women friendly.

**Supplementary Information:**

The online version contains supplementary material available at 10.1186/s12885-024-12223-8.

## Background

Worldwide, cervical cancer is the fourth most common cancer among women and mainly affects women in low- and middle-income countries. Vaccination against the causative human papillomavirus (HPV) and screening and treatment of pre-cancerous lesions are effective strategies in cervical cancer prevention [1]. Women vaccinated with either the bivalent or quadrivalent vaccine at ages 11–15 years might be screened on three occasions (approximately at ages 30, 40, and 55 years), and women vaccinated with the nonavalent vaccine might be screened twice in their lifetime [2].

Herd protection might reduce the need for more intensive screening in unvaccinated members of vaccinated cohorts [3]. However, screening remains essential for preventing cervical cancer in older unvaccinated cohorts. Increased uptake in HPV vaccination is likely to lead to increased screening intervals, improved adherence to the screening schedule and better access to the screening services resulting into increased uptake of the screening services and a reduction in cervical cancer.

The World Health Organization’s (WHO) elimination strategy recommends high risk human papillomavirus (hrHPV) self-collected testing as primary screening because it is very sensitive, objective and highly accepted among women. It is a promising method to increase screening uptake [1].

Uganda has one of the highest incidence and mortality rates due to cervical cancer. This has been attributed to low HPV vaccination and screening uptake which in turn are influenced by lack of knowledge about cervical cancer coupled with a poorly functioning health infrastructure [4, 5] and high prevalence of risk factors like HIV among others [6]. The incidence of cervical cancer rose at a rate of 1.8% per year over a period of 20 years between 1991 to 2010 [7]. Incidence of cervical cancer was projected to increase by 35.3%, and Age Standardized Incidence Rates projected to increase up to 66.1 per 100 000 women by the year 2030, [8] if no intervention is put in place.

Cervical cancer is a reproductive cancer, generally caused by persistent infection with hrHPV. This virus is sexually transmitted and therefore prevention of cervical cancer is partly dependent on male support and involvement. Men play an important role in HPV transmission as HPV DNA has been detected in their genitalia ranging from 55.3—76.6% among HIV positive men and 38.6—47.6% among HIV negative men [9]. Furthermore, men are often decision makers in rural settings in Uganda and will often decide whether their women can be screened [10] and treated for pre-cancerous lesions [11].

Limited knowledge among men about the risk factors and causes of cervical cancer can limit uptake of screening. Increased knowledge among men would enable them to embrace the prevention programs and also to support and encourage the women to go for screening and to seek medical help on acquisition of symptoms of cervical cancer [12].

Poor recognition of cancer symptoms by both patients and primary healthcare professionals may contribute to the advanced stage at diagnosis and poor survival of cervical cancer patients in Uganda [13, 14]. Ugandan women with vaginal symptoms often first attribute their symptoms to gynaecological infections such as candidiasis and sexually transmitted infections. Only if symptoms persist despite medication, then women sometimes attribute their symptoms to cancer [15]. The presence of symptoms usually implies advanced disease. Early recognition will aid in early diagnosis before cancer is developed.

Knowledge of the cause, risk factors and symptoms of cervical cancer are determinants of preventive behavior such as HPV vaccination and screening, it also promotes seeking medical help for early detection, and these translate into reduction in the incidence, morbidity and mortality due to cervical cancer [16]. Information is needed on how best to design preventive interventions in the community. Prevention of the cancer is key in the fight towards elimination.

The Prevention and Screening Innovation Project Towards Elimination of Cervical cancer (PRESCRIP-TEC) [17] was initiated to evaluate the feasibility of the WHO strategy in resource-constrained circumstances and targets women living in rural areas in Uganda, Bangladesh, Slovakia republic and India.

As part of the PRESCRIP-TEC project, the primary objective of this study was to gain understanding of the baseline cervical cancer knowledge and intended behavior towards screening and treatment among women and men/decision makers in a rural community setting in Uganda. The secondary objective was to enhance the specific communication strategy aimed at increasing uptake for cervical cancer screening and seeking help for early detection.

## Methods

### Study setting

The study was conducted from March 2022 to July 2022 in the districts of Kakumiro and Kagadi in Western Uganda. In both districts, 3 sub-counties were targeted in Kakumiro district and in Kagadi district.

Each of the sub-counties is served by at least one Health Centre III (HC III). HC III is the lowest level facility in the health structure where cervical cancer screening and treatment is carried out as per the national guidelines and Strategic plan 2010- 2014 [18].

### Study population and eligibility criteria

Women were included if they were aged 30–49 years old, able to participate in a screening program, able to give informed consent for participation in the study and had the skills to respond to questions. The age limits align with the Uganda national cervical cancer screening guidelines. Women were excluded if they were pregnant, had history of hysterectomy, had prior treatment for cervical disease or had signs and symptoms of cervical cancer.

Men (husbands/decision makers of the women from the same households) aged 20 – 70 years with skills to respond to questions and or engage in an interview were included. Men who refused or were unable to provide informed consent were excluded.

### Study design and sampling

This was a cross-sectional study and sampling was non-randomised. The sub-counties in Kakumiro district were purposively selected for having no cervical cancer screening services available while those in Kagadi district were randomly selected because screening was already available prior to implementation of the PRESCRIP-TEC project. The sample size was arrived at by calculating differences in awareness scores of at least 10% between women and men, applying a two-sided two-sample test with alpha = 0.05, a standard deviation of 0.2 and allowing for a 15% non-response rate. We targeted 400 women and their male counterparts from each of the districts.

### Data collection

The Village Health Teams (VHTs) provide the first level of primary health care in Uganda. The teams consist of community volunteers, both women and men, chosen by community members to provide healthcare services to their communities and are regarded as Health Centre I (HC I) [19–21]. Each VHT serves about 1000 people or 25 households in a village [22–24], and they provide linkage between the community and the Health Centres [19, 20, 23]. In addition to other functions, the VHTs are to raise awareness and mobilise community members to participate in cervical cancer prevention activities [18]. For each sub-county prior to data collection, 12 VHTs were trained for a period of 5 days by the principal investigator (CN). The training included the basics of cervical cancer, the cause, risk factors, symptoms, signs and prevention of cervical cancer. VHTs were also trained on the research protocol, Good Clinical Practice, the consenting process and the questionnaire. VHTs were selected by the Head VHT based on literacy levels. They collected data from one village at a time until; all homesteads were visited. All interviews were conducted in the homes of the women and her husbands/decision makers after establishing eligibility and informed consent obtained. Data was initially collected on paper questionnaires and later double entered into the REsearch Data Capturing system (REDcap) by data entry clerks. Data was checked for completeness and as part of quality control, the research assistants revisited the households when data was not complete and or aspects of the data were not clear.

### Instruments

In order to measure cervical cancer awareness and intended behavior, the adapted version of the African Women Awareness of CANcer (AWACAN) instrument was administered. The AWACAN tool has shown to be reliable and valid for use in Sub-Saharan Africa. It is a 41-item questionnaire that measures women’s awareness of cervical cancer. The tool measures awareness in the following domains: risk factors, symptoms, lay beliefs, confidence in appraisal, health-seeking behaviors, and barriers to health care and has socio-demographic questions [25]. The questionnaire was validated with questions on knowledge of risk factors and symptoms of cervical cancer, help-seeking behavior, confidence skills and behavior regarding symptoms and barriers to seeking help. Prior to data collection, the instrument was pilot tested and forward and backward translation to Runyoro-Kitara language done. Based on the pilot, separate versions for women and their husbands or household decision makers were prepared.

The AWACAN questionnaire assessed 11 risk factors and 12 symptoms of cervical cancer. Knowledge of risk factors was only assessed on respondents who had ever heard of cervical cancer while symptoms were assessed on the total sample included as per the AWACAN tool.

To assess intended behavior, we measured help-seeking behavior, confidence in identifying signs and symptoms and barriers to seeking medical help. Help seeking behavior was assessed using 6 questions. Confidence in identifying signs and symptoms of cervical cancer was measured using 3 questions. Barriers to seeking medical help was measured using 12 questions.

### Data analysis

The collected data was coded, entered and analysed using Statistical Package for Social Sciences version 26.

Univariate analysis was done to obtain the descriptive statistics (demographic characteristics) of the participants. Bivariate analysis and comparison of all AWACAN question responses between female respondents and men was done. Measures on knowledge scores included median and interquartile ranges. Knowledge scores were categorised into binary outcome as less knowledgeable below the median scores and highly knowledgeable with median and above the median scores. Bivariate analysis using Chi square tests were conducted to determine associations between the binary knowledge scores on risk factors and symptoms of cervical cancer with socio-demographic correlates. Multivariate logistic regression models were applied on categorical variables to determine the magnitude of association between knowledge of risk factors and symptoms of cervical cancer with the demographic factors, and checked for assumptions.

Statistical significance was set at *p*-value < 0.05.

## Results

### Socio-demographic characteristics of the participants

A total of 1,416 participants, 724 (51.1%) women and 692 (48.9%) men were interviewed. More than half of them were less than 40 years old with a mean age of 37 years for the female respondents and 38 years for the male respondents (Table 1).

**Table 1:** Sociodemographic characteristics of participants

**Variable**	**Category**	**Female *N* = 724**	**Male *N* = 692**
Age (years)	Mean (SD)	37.39 (5.69)	37.88 (7.13)
		**n (%)**	**n (%)**
Education	No education	69 [16.2]	71 [16.1]
	Primary School	325 [76.5]	335 [75.7]
	Secondary school	31 [7.3]	33 [7.5]
	Tertiary	0	3 [0.7]
Marital status	Married	312 [63.3]	272 [62.0]
	Single	48 [9.8]	56 [12.8]
	Widowed	17 [3.4]	13 [3.0]
	Cohabiting	107 [21.7]	90 [20.5]
	Divorced	9 [1.8]	8 [1.7]
Ever heard of cervical cancer?	Yes	510 [71.0]	459 [67.2]
	No	208 [29.0]	224 [32.8]
Has a health worker ever tested you for cervical cancer?	Yes	61 [8.8]	NA
	No	633 [91.0]	NA
	Refused	1 [0.1]	NA
	Don’t know	1 [0.1]	NA
Who makes decisions concerning your health?	Myself	229 [31.7]	NA
	My partner	254 [35.2]	NA
	Myself and partner jointly	226 [31.8]	NA
	Someone else in the household	11 [1.5]	NA
	Myself and someone else jointly	2 [0.3]	NA

### Knowledge on risk factors of cervical cancer

Knowledge of risk factors for cervical cancer was assessed in 510 women and 459 men. These are participants who responded positively to ever having heard of cervical cancer. HPV infection being a risk factor for cervical cancer was known by 74.5% of the women and 72.2% of the men. Having many sexual partners was recognised by 82.9% women and 78.8% men, while smoking was recognised by only 55.6% of the women and 54.5% of the men. Giving birth to more than 3 children was the only risk factor with significant difference in knowledge between men and women; the women were more knowledgeable than the men. See details in Supplementary Material Table 1.

### Knowledge on symptoms of cervical cancer

Knowledge on symptoms and/or signs of cervical cancer was assessed in 724 women and 692 men. A persistent smelly vaginal discharge was the commonest recognised symptom of cervical cancer while persistent diarrhoea was the least recognised symptom. The women were 8 times more likely to recognise lower back pain as a symptom of cervical cancer than the men. See details in Supplementary Material Table 2.

### Knowledge scores on risk factors and symptoms

A total of 510 women and 459 men were assessed on 11 risk factors for cervical cancer. The median number of risk factors recognised was 8 (IQR 6—10) for both women and men; 60% of the women and 57.4% of the men could recognise 8—11 risk factors, 22 (4.3%) of the women and 33 (7.2%) of the men couldn’t recognise a single risk factor for cervical cancer.

Of the 724 women and 692 men assessed on recognition of symptoms, 64.6% and 60.5% respectively could recognise 8—12 symptoms out of the 12 symptoms, while 67 (9.3%) women and 71(10.3%) men couldn’t identify a single symptom of cervical cancer Table 2.

**Table 2:** Knowledge scores of cervical cancer risk factors and symptoms

Knowledge scores	Females		Males	
**Variables**	**Risk factors = 510**	**Symptoms *n* = 724**	**Risk factors *n* = 459**	**Symptoms *n* = 692**
Median score [8], IQR [6.0 – 10.0]	65 (12.8%)	87 (12.0%)	43 (9.4%)	64 (9.2%)
Below median score	202 (39.6%)	256 (35.4%)	196 (42.7%)	273 (39.5%)
Above median score	243 (47.6%)	381 (52.6%)	220 (47.9%)	355 (51.3%)

*Key*: *IQR* Interquartile range

### Association between knowledge of risk factors and symptoms of cervical cancer with social demographic characteristics

Age, education attainment and marital status were all not associated with knowledge of risk factors and symptoms for cervical cancer for both women and men. See Supplementary Information Tables 3 and 4.

### Decision making in comparison with other factors

Women whose husbands/decision makers made decisions for them with regard to issues of health were 15 times more likely to be less knowledgeable about risk factors of cervical cancer compared to women who made their own decisions or in partnership with their husbands. They were also more likely to be older and less or not educated (Table 3).

**Table 3:** Decision making in comparison with other factors

Variable	Category	Myself n (%)	Partner n(%)	Myself and Partner n (%)	X^2^	*P* Value
Risk factors	Low knowledge	52 (32.9)	74 (45.1)	66 (37.7)	14.54	0.001
	High knowledge	108 (67.18)	90 (54.9)	109 (62.3)		
Symptoms	Low knowledge	48 (27.9)	52 (29.4)	64 (33.0)	2.11	0.72
	High knowledge	124 (72.1)	125 (70.6)	130 (67.0)		
Age	30—39	61 (79.2)	59 (65.6)	61 (55.5)	17.33	0.002
	40—49	16 (20.8)	31 (34.4)	49 (44.5)		
Education	CP	20 (12.5)	12 (7.3)	25 (14.3)	36.05	0.003
	CS	10 (6.3)	4 (2.4)	6 (3.4)		
	LP	42 (26.3)	56 (34.1)	60 (34.3)		
	None	5 (3.1)	18 (11.0)	20 (11.4)		

*Key*: *CP* Completed Primary school, *CS* Completed secondary school, *LP* Didn’t complete primary school, *None* No formal education, *X*^*2*^ Chi square

### Knowledge of risk factors, symptoms and intended behavior towards cervical cancer among women and men

There was no significant difference between the women and men in their knowledge of risk factors and symptoms. However, men had better health seeking behavior than women, and more confidence, skills and behavior towards signs and symptoms of cervical cancer. See details in Supplementary Table 5.

Barriers to seeking medical help were found in 316 (43.9%) of the women and 314 (45.4%) men, however, there was no difference between the two groups, neither did barriers affect them significantly. See details in Table 4 and Supplementary Table 5.

**Table 4:** Knowledge of risk factors, symptoms of cervical cancer and intended behavior among men and women

**Variable**	**Category**	**Females** **n [%]**	**Males** **n [%]**	**X**^**2**^	***P*-value**	OR	95% CI
Knowledge of cervical cancer risk factors *n* = 969	Low score	154 [30.2]	196 [42.7]	0.03	0.86	0.98	0.74—1.292
	High score	356 [69.8]	263 [57.3]				
Knowledge of cervical cancer symptoms *n* = 1416	Low score	256 [35. 4]	273 [39.5]	2.53	0.11	1.19	0.96—1.48
	High score	468 [64.6]	419 [60.5]				
Help seeking behavior *n* = 1409	No	313 [43.6]	161 [23.30]	64.96	** < 0.001**	0.393	0.312 -0.495
	Yes	405 [56.4]	530 [76.7]				
Confidence skills and behavior to a sign or symptom *n* = 1407	No	221 [30.9]	130 [18.8]	27.28	** < 0.001**	0.52	0.40 -0.67
	Yes	495 [69.1]	561 [81.2]				
Barrier to seeking help *n* = 1411	No	404 [56.1]	377 [54.6]	0.34	0.56	1.07	0.86—1.31
	Yes	316 [43.9]	314 [45.4]				

*Key*: *OR* Odd’s ratio, *CI* Confidence Interval

## Discussion

This community-based study provides information on baseline knowledge of risk factors and symptoms of cervical cancer and intended behavior towards screening and treatment for cervical cancer of women and men living in rural Uganda. We found that knowledge of risk factors and symptoms was high in the majority of participants, for both women and men. We also found that despite this high knowledge, intended behavior to screen and the apparent insignificant barriers, the screening uptake was very low necessitating further investigation into this discrepancy.

Results from this study can inform specific target group communication and other implementation strategies on cervical cancer prevention aimed at increasing uptake for screening and early detection.

### Knowledge on cervical cancer

In our study, there was no association between the demographic factors and knowledge of risk factors and symptoms, probably because the population was generally uniform with no significant differences with regard to their demographic characteristics and knowledge of cervical cancer. This is unlike other studies where low knowledge of risk factors was associated with not being married [26] and low education [27]. In another study the level of education was associated with high knowledge for risk factors while older age was associated with symptom awareness [28].

More than 60% of both women and men were able to recognize at least 8 out of 11 and 12 risk factors and symptoms respectively of cervical cancer, making it a knowledgeable population with no difference between the women and men. Knowledge of risk factors promotes preventative behavior like screening and vaccination against HPV while knowledge of the symptoms promotes health seeking behavior and thus early detection.

It is known that knowledge of cervical cancer is a determinant of screening uptake [5, 29], but the knowledge of the participants in this study was not commensurate with the level of uptake. This reciprocates with the review by Lott et al. where they analysed interventions to increase uptake of cervical screening in Sub-Saharan Africa (SSA) and found that educational interventions were the most common type of intervention used to increase uptake of cervical screening in SSA but also the least effective [30]. However, a few studies that utilised peer health educators and community health educators as part of the implementation strategy were an exception emphasising the role of social ties, using educators that were already known to the study participants [31, 32]. This means that education as an intervention may not yield much, but if delivered by peers and community health educators it may influence screening uptake. In addition, education needs to be combined with actual availability of the appropriate services. This was noted in our study areas where knowledge of the respondents was high in both districts, but uptake was 6.6% in the district with no services and 11.1% in the district with services. This difference was not significant but notably more women had been screened in the latter and the few women who had been screened in the former could have sourced it in another district.

### Intended behavior

The Integrated Behavioral Model (IBM) is hinged on the fact that the most important determinant of behavior is intention to perform that behavior, and that intention is influenced by attitudes, perceived norms and personal agency [33]. In our study the high knowledge and good intended behavior did not translate into screening behavior. Lott et al. observed very high willingness to screen but that intent to screen did not always translate into uptake of cervical screening [30]. Similarly, Ndikom et al., described an increase from 75.8% to 91% in willingness to screen, yet no change in actual screening was observed [34]. Therefore, intended behavior alone does not guarantee behavior change. More research is required to find out what more needs to be done to allow this intention to translate into actual screening uptake.

### Decision making

In this study, 60% of the women had decisions made for them by the men or in partnership with them. We found that the women who took no part and depended on their husbands to make decisions concerning their health were least knowledgeable about risk factors of cervical cancer, followed by those who decided in partnership with their husbands, while the women who made their own decisions were most knowledgeable of all the women.

In addition, women whose husbands made decisions for them were least educated or mostly with no formal education at all and were also older putting them at most risk for cervical cancer [35]. The trend was similar in those who made decisions in partnership with their husbands and were next at risk. The women who made their own decisions were more knowledgeable on risk factors, most educated and younger than the two groups. This is in agreement with another study on women’s healthcare decision making and cervical cancer screening uptake done in 4 countries in SSA, which showed that women who are able to make autonomous healthcare decisions were most likely to uptake cervical cancer screening followed by those who decided in partnership with their husbands and least of all were those whose decisions were solely made by their husbands [36].

Ndikom et al., [34] in their study in Nigeria reported lack of decision-making ability as a barrier to screening. Formal education and training were found to be key to raising women’s confidence as was reported in the Wagner study [37]. Building confidence in those women was demonstrated to have a multiplying effect in getting women to screen. In their pilot, women who had screened were trained as advocates for cervical cancer screening and this resulted in increased numbers of women taking up screening [37]. Therefore, policy interventions should focus on empowering women to make autonomous decisions or in partnership with their husbands with regard to issues concerning health.

### Male involvement

HPV is a very common sexually transmitted infection affecting 80% of adults by age 45 [38]. Men play a role in the transmission of the HPV infection. While they also suffer from HPV related cancers, currently these are not screened for and the virus not treated once contracted. HPV vaccination prevents new infections and works best before any exposure to HPV. This makes HPV vaccination for unexposed males essential [38]. At the moment this is way out of reach for low-income countries like Uganda but it is important to incorporate this message in the education packages for men. It is important for men to know that cervical cancer is not a disease of women only, but men need to protect themselves from HPV infection in order to protect their wives. If demand is created it is hoped that the numbers may bring down the costs of the vaccine in the long run, which is the biggest hindrance. Men also need to support their daughters to get vaccinated and their sons when resources permit. De Fouw et al., reported in their study that men were willing to have their daughters vaccinated against HPV [12]. This makes men a critical target for impactful communication and other interventions aimed at increasing vaccination and screening uptake. Men and their involvement in the control of cervical cancer are key players in the elimination of cervical cancer [11–13]. In this study men had significant better help seeking behavior compared to the women. This could be attributed to the fact that health seeking is associated with certain expenses such as transport and medical costs. Since men generally are the bread earners and decision makers [11], they usually control the budget to spend rather than the women. If men are better educated about risk factors and symptoms of cervical cancer, they are more willing to support their wives in providing support for cervical cancer screening. This is supported in various studies where men were willing to support their wives to seek help in case of symptoms and also willing to support them for screening [13, 39–41]. Other studies have tried integrating male services into cervical cancer screening programs with some positive results [30]. This could be an area for further research.

Additionally, men felt more confident at recognising a symptom of cervical cancer than the women. This could be attributed to a cultural norm where the men are the decision makers and thus expected to know more than the women, or a possible socially desirable response.

Increasing community knowledge of cervical cancer symptoms among others and tackling perceived barriers to health seeking, could lead to prompt and appropriate symptom appraisal and help seeking and contribute to improved cancer outcomes.

Given more education on cervical cancer and the role they can play in preventing it, combined with targeted communication messages and active involvement, men would significantly impact on screening uptake which would in turn contribute towards elimination of cervical cancer.

### Barriers

Transport as a barrier to seeking medical help did not feature significantly but more than 40% of the women reported it was a problem. Therefore, economic empowerment of the women so that they do not depend on the men, could possibly influence them to taking up screening for cervical cancer since lack of transport has been cited to be a barrier to screening in several studies [29]. However, economic incentives to women increased uptake very minimally of less than 20% [39, 42]. This intervention may not work and let alone be sustainable. It may need to be coupled with other interventions.

Barriers to seeking medical help were surprisingly lower in our study compared to other studies probably because the respondents were aware of most of the risks and symptoms of cervical cancer and the advantage of seeking medical help. More than half of the participants both women and men would seek medical help once there are symptoms. This is in contrast with the study by Birhanu et al., [43] where cervical cancer was associated with a lot of stigmas. It was thought that frequent sex with multiple partners was the cause. Afraid of stigma, women shied away from seeking help. Issues like long waiting time, fear of the diagnosis, lack of transport and others did not feature as obstacles to seeking medical help in more than half of the participants in our study contrary to what was previously reported [29].

### Service availability

In the review by Lott et al., studies that utilised innovative service delivery approaches focusing on availability, accessibility and appropriateness of screening services for women resulted in the greatest increases in screening uptake [30]. At the time of this study, there were no screening services in the entire district which would explain the low uptake confirming availability of services to be crucial in enabling behavior to screen [34, 44].

In another study, accessibility was addressed by changing location of the screening services from health facilities and bringing them to the doorsteps of the women avoiding transport as a barrier. This led to tremendous increase in screening uptake which was not possible when women were referred to hospital for screening [39]. Community based service delivery addressed the issue of accessibility. We recommend this approach as it has been proved to yield increase in screening uptake.

Uptake was even higher when the test was switched from Visual Inspection with Acetic acid (VIA) to self-collection. In Uganda and other places, self-collection registered uptake higher than 90% and the women reported very good attitude towards this type of screening. Coupled by integration into existing services and having community health workers drive the project was acceptable by the women because of working with their own well known community healthcare workers and feeling in charge of their own health given the technique of self-collection in the comfort and privacy of their homes. This made the service delivery women friendly/appropriate and resulted into screening uptake of more than 90% [45]. We therefore recommend this proven women-friendly approach of self-collection in their homes by community health workers and anticipate an increase in the screening uptake in this community.

### Next steps

In the next steps we provide communication messages geared towards cervical cancer, risk factors, signs and symptoms and its’ prevention resulting from this study and specific to women and men/decision makers for our mobilisation, health education and counselling strategies. We also highlight the role of men in cervical cancer prevention. This will be followed by providing the screening services of HPV DNA self—collection at the doorsteps of eligible women driven by the same community workers who carried out this survey in the PRESCRIP-TEC feasibility study on uptake of screening. These interventions will be replicated in 3 other countries namely Bangladesh, India and Slovakia under the PRESCRIP-TEC project.

### Strengths and limitations

This study was conducted by trained local VHTs who spoke the local language and knew the homes of all the residents ensuring coverage of the area. The willingness of men who are decision makers to be involved in the prevention and early detection of cervical cancer for their wives is an advantage for the next phase of intervention of this study. Another advantage was the fact that the study was conducted in a very rural place which may be similar and may be representative of many of the other parts of the country in Uganda. The other strength of this study is that we investigated the intended behavior of the men which greatly informs policy on intervention. Using a standardised tool the AWACAN adds validity to our results, the findings of which can be used to develop target specific messages on cervical cancer prevention and early detection.

The sample size of 1,416 participants was less than was calculated due to the fact that the study was conducted during the rainy season, making it sometimes difficult to find potential participants. This however has enough power to draw conclusions from the results generated.

Among the limitations, social desirability cannot be ruled out given that the questionnaires were administered face to face. This was minimised by the fact that the VHTs were adequately trained and emphasised to the participants that their responses were going to be kept anonymous with confidentiality and privacy observed.

There was a degree of selection bias given that the sub-counties in Kakumiro district were selected by community and political leaders on the basis that there were no screening services in those areas. However, we think that results would have been similar if another area had been selected for the study within the same region. Selection bias was minimised by ensuring that all villages and all households with eligible participants were included in the study with the help of the local VHTs.

We were unable to compare the study participants and those who refused to participate with respect to age, education and other characteristics due to protocol restriction.

## Conclusion

Knowledge of risk factors, symptoms and signs of cervical cancer in the majority of participants was high in both women and men without significant difference between the two groups. The intended behavior for both women and men towards screening or early diagnosis and early detection was generally positive. Barriers to seeking medical help did not significantly affect the population to go for screening and yet despite all the above factors, screening uptake was very low. Notably, lack of decision making for women was significantly associated with low knowledge, low or no formal education and increased age.

In this study we noted that the high knowledge and intention to screen alone, did not translate into increased uptake. However, if combined with other interventions like communication strategies specifically targeting the men and women, male involvement, women empowerment and availability of community-based women friendly services among others, could help to translate into increased screening uptake. Further research on this is warranted. Knowledge alone does not translate to screening uptake, but a combination of approaches should be the focus of any communication strategy towards elimination of cervical cancer.

### Supplementary Information

**Supplementary Material 1. **

## Data Availability

The datasets generated and/or analysed during the current study are available in the open data source depository under DataverseNL. The registration number is 10.34894/LO4AA6. @data{LO4AA6_2024, author = {Koot, Jaap and Schans, Jurjen van der and Zeeuw Janine de}, publisher = {DataverseNL}, title = {{Prevention and Screening Innovation Project Towards Elimination of Cervical Cancer}}, year = {2024}, version = {DRAFT VERSION}, doi = {10.34894/LO4AA6}, url = {10.34894/LO4AA6}.

## Abbreviations

HPV: Human papillomavirus
hrHPV: High risk human papillomavirus
PRESCRIP-TEC: Prevention and screening innovation project towards elimination of cervical cancer.
SSA: Sub-Sahara Africa
VHT: Village health teams
WHO: World Health Organization
