# Supplementary material for: Baseline knowledge on risk factors, symptoms and intended behavior of women and men towards screening and treatment of cervical cancer in rural Uganda: a cross-sectional study
Source: BMC Cancer. 2024 Apr 11;24:450. doi: 10.1186/s12885-024-12223-8 (PMC11008004; doi:10.1186/s12885-024-12223-8)
Supplement: Supplementary file 1 — Supplementary Material 1. [file 12885_2024_12223_MOESM1_ESM.docx]

**Supplementary Material**

**Supplementary material Table 1: Knowledge/recognition of risk factors of cervical cancer**

| **Variable** | Category | Females n [%] | Males n [%] | X^2^ | P Value |
| --- | --- | --- | --- | --- | --- |
| Getting a sexually transmitted infection called Human Papilloma virus [HPV] | Yes | 377 [ 74.5] | 328 [ 72.2] | 0.92 | 0.63 |
|  | No | 30 [5.9] | 33 [ 7.3 ] |  |  |
|  | Don’t know | 99 [19.6] | 93 [20.5] |  |  |
| HIV/AIDs | Yes | 304 [60.6] | 275 [61.2] | 1.03 | 0.60 |
|  | No | 72 [14.3] | 72 [16.0] |  |  |
|  | Don’t know | 126 [ 25.1] | 102 [22.7] |  |  |
| Infection with other sexually transmitted diseases apart from HIV and HPV | Yes | 359 [71.1] | 317 [69.7] | 0.31 | 0.86 |
|  | No | 38 [7.5] | 38 [8.4] |  |  |
|  | Don’t know | 108 [21.4] | 100 [22.0] |  |  |
| Using birth control pills/family planning for more than 5 years | Yes | 374 [74.1] | 331 [72.9] | 0.17 | 0.92 |
|  | No | 44 [ 8.7] | 42 [ 9.3] |  |  |
|  | Don’t know | 87 [17.2] | 0 |  |  |
| Having un-protected sex | Yes | 369 [72.9] | 341 [75.3] | 0.94 | 0.63 |
|  | No | 52 [10.3] | 46 [10.2] |  |  |
|  | Don’t know | 85 [16.8] | 66 [14.6] |  |  |
| Smoking cigarettes | Yes | 282 [55.6] | 248 [54.5] | 0.41 | 0.82 |
|  | No | 121 [23.9] | 106 [23.3] |  |  |
|  | Don’t know | 104 [20.5] | 101 [22.2] |  |  |
| Having a sexual partner who is not circumcised | Yes | 337 [67.8] | 333 [73.2] | 3.96 | 0.14 |
|  | No | 49 [9.9] | 43 [9.5%] |  |  |
|  | Don’t know | 111 [22.3] | 79 [17.4%] |  |  |
| Having sex before the age of 17 | Yes | 375 [75.2] | 339 [74.5] | 1.00 | 0.61 |
|  | No | 47 [9.4] | 37 [8.1] |  |  |
|  | Don’t know | 77 [15.4] | 79 [17.4] |  |  |
| Giving birth to three or more children | Yes | 316 [62.8] | 255 [ 56.3] | 6.41 | **0.04** |
|  | No | 87 [17.3] | 107 [23.6] |  |  |
|  | Don’t know | 100 [19.9] | 91 [20.1] |  |  |
| Having many sexual partners | Yes | 417 [82.9] | 358 [78.8] | 2.89 | 0.24 |
|  | No | 31 [6.2] | 38 [8.3] |  |  |
|  | Don’t know | 55 [10.9] | 59 [12.9] |  |  |
| Not going for regular screening/testing for cervical cancer | Yes | 351 [69.6] | 314 [68.9] | 0.39 | 0.82 |
|  | No | 79 [15.7] | 78 [17.1] |  |  |
|  | Don’t know | 74 [14.7] | 64 [14.0] |  |  |

Supplementary material **Table 2: Knowledge of Symptoms of cervical cancer**

| **Variable** | Category | Females n [%] | Males n [%] | X^2^ | P value |
| --- | --- | --- | --- | --- | --- |
| Vaginal bleeding between menstrual periods | Yes | 495 [69.1] | 483 [70.3 ] | 0.44 | 0.80 |
|  | No | 63 [8.8] | 54 [7.5] |  |  |
|  | Don’t know | 158 [22.1] | 151 [21.9] |  |  |
| Persistent lower back pain | Yes | 522 [72.4] | 469 [68.3] | 7.73 | **0.02** |
|  | No | 48 [6.7] | 74 [10.8] |  |  |
|  | Don’t know | 151 [20.9] | 144 [21.0] |  |  |
| A persistent smelly vaginal discharge | Yes | 538 [74.9] | 499 [72.6] | 1.21 | 0.55 |
|  | No | 57 [7.9] | 55 [8.0] |  |  |
|  | Don’t know | 123 [17.2] | 133 [ 19.4] |  |  |
| Discomfort or pain during sex | Yes | 533 [74.1] | 478 [69.6] | 4.00 | 0.14 |
|  | No | 48 [6.7] | 47 [6.9] |  |  |
|  | Don’t know | 138 [19.2] | 161 [23.5] |  |  |
| Menstrual periods that are longer or heavier than usual | Yes | 525 [73.3] | 484 [ 70.7] | 2.00 | 0.37 |
|  | No | 54 [7.5] | 49 [7.2] |  |  |
|  | Don’t know | 137 [19.2] | 152 [22.2] |  |  |
| Persistent diarrhoea | Yes | 213 [29.7] | 196 [28.6] | 0.34 | 0.84 |
|  | No | 298 [41.6 ] | 295 [43.0] |  |  |
|  | Don’t know | 206 [ 28.7] | 195 [28.4] |  |  |
| Vaginal bleeding after menopause | Yes | 507 [ 70.4 ] | 452 [ 66.3] | 2.97 | 0.23 |
|  | No | 68 [9.4] | 69 [10.1] |  |  |
|  | Don’t know | 145 [20.2] | 161 [23.6] |  |  |
| Persistent lower abdominal/pelvic pain | Yes | 481 [ 67.0] | 464 [ 67.6] | 0.45 | 0.80 |
|  | No | 79 [11.0] | 68 [9.9] |  |  |
|  | Don’t know | 158 [ 22.0] | 154 [22.4 ] |  |  |
| Vaginal bleeding during or after sex | Yes | 516 [ 71.7] | 488 [71.2] | 3.11 | 0.21 |
|  | No | 59 [8.2] | 42 [6.1] |  |  |
|  | Don’t know | 145 [20.1 ] | 155 [22.6 ] |  |  |
| Blood in urine or stool | Yes | 366 [51.7] | 353 [51.7] | 0.10 | 0.95 |
|  | No | 142 [20.1] | 141 [20.6] |  |  |
|  | Don’t know | 200 [28.2 ] | 189 [27.7] |  |  |
| Unexplained weight loss | Yes | 387 [54.2 ] | 384 [55.8 ] | 0.64 | 0.73 |
|  | No | 139 [19.5] | 123 [17.9] |  |  |
|  | Don’t know | 188 [26.3 ] | 181 [26.1] |  |  |
| Itching in the vagina | Yes | 493 [69.8] | 464 [68.3] | 0.96 | 0.62 |
|  | No | 87 [12.3] | 80 [11.8] |  |  |
|  | Don’t know | 126 [17.8 ] | 135 [19.9] |  |  |

**Supplementary material Table 3: Correlation between social demographics and knowledge of risk factors**

| **Variable** | **Females N = 288**  **n [%]** | | | | | **Males N = 288**  **n [%]** | | |  |
| --- | --- | --- | --- | --- | --- | --- | --- | --- | --- |
| Variable | Low Knowledge | High Knowledge | | | COR  [ 95% CI] | Low Knowledge | High Knowledge | COR [ 95% CI] | |
| **Age** | | | | | | | | | |
| 20 - 29 | 0 | 0 | | | 0 | 2 (0.7) | 10(3.5) | ref | |
| 30 - 39 | 71(24.7) | 117  (40.8) | | | ref | 55  (19.2) | 120  (35.5) | 4.00  (0.67-23.73) | |
| 40 - 49 | 33 [11.5] | 66 [23.0] | | | 0.82  [0.49 - 1.37] | 47  [16.4] | 53 [18.5] | 1.484  [0.55 - 3.98] | |
| 50 - 70 | 0 | 0 | | | 0 | 8  [2.8] | 10  [3.5] | 0.90  [0.33 - 2.48] | |
| **Education** | | |  |  | | | | | |
| No education | 21  [7.3] | 24 [8.3] | | | ref | 21  [7.3] | 21  [7.3]] | ref | |
| Primary school | 77  [26.7] | 145  [50.3] | | | 0.46  [0.15 - 1.39] | 82  [28.7] | 142 [49.7] | 1.00  [0.06 - 17.07] | |
| Secondary School | 6  [2.1] | 15  [5.2] | | | 0.75  [0.28 - 2.02] | 8  [2.8] | 10  [3.5] | 1.71  [ 0.11 - 27.72] | |
| Tertiary level | 0 | 0 | | | 0 | 1  [0.3] | 1  [0.3] | 1.25  [0.07 - 23.26] | |
| **Marital status** | | |  |  | | | | | |
| Married | 64  [22.3] | 122  [42.5] | | | ref | 71  [24.8] | 111  [38.8] | ref | |
| Not married | 40  [13.9] | 61  [21.3] | | | 1.26  [0.76 - 2.08] | 40  [14.0] | 62 [21.7] | 1.54  [0.09 - 25.49] | |

**Supplementary material Table 4**: **Knowledge of cervical cancer symptoms and demographic correlates.**

| **Variable** | **Females N = 423**  **n [%]** | | | **Males N = 442**  **n [%]** | | |  |
| --- | --- | --- | --- | --- | --- | --- | --- |
|  | Low Knowledge  n [%] | High knowledge  n [%] | COR  [95% CI] | Low knowledge  n [%] | High knowledge  n [%] | COR  [95% CI] |  |
| **Age** |  | | |  | | |  |
| 20 - 29 | 0 | 0 | 0 | 9  [2.0] | 15  [3.4] | ref |  |
| 30 - 39 | 79  [18.7] | 189 [44.7] | ref | 86  [19.5] | 161 [36.4] | 0.98  [0.31 - 3.06] |  |
| 40 - 49 | 52 [12.3] | 103 [24.3] | 1.20  [ 0.79 - 1.84] | 51  [11.5] | 93  [22.0] | 1.10  [0.48 - 2.51] |  |
| 50 - 70 | 0 | 0 | 0 | 10  [2.1] | 17  [3.8] | 1.07  [0.46 - 2.52] |  |
| **Education attainment** | | | | | | | |
| No education | 23 [5.4] | 46 [10.8] | ref | 31  [7.0] | 40 [9.0] | ref |  |
| Primary school | 102 [24.1] | 222 [52.4] | 0.48  [0.17 - 1.33] | 110  [24.9] | 225  [50.9] | 0.65  [0.06 - 7.45] |  |
| Secondary School | 6  [1.4] | 25 [5.9] | 0.52  [0.21 - 1.31] | 13  [2.9] | 20  [4.5] | 1.02  [0.09 - 11.40] |  |
| Tertiary level | 0 | 0 | 0 | 1  [0.2] | 2  [0.5] | 0.77  [0.06 - 9.37] |  |
| **Marital Status** | | | | | | | |
| Married | 87  [20.6] | 189  [44.7] | ref | 93  [21.1] | 178 [40.4] | ref |  |
| Not married | 44  [10.4] | 103  [24.3] | 0.93 [ 0.60 - 1.43] | 60  [13.6] | 107 [24.3] | 3.83  [0.34 - 42.77] |  |

**S**upplementary material **Table 5: Bivariate analysis comparing female and male respondents on intended behaviour towards cervical cancer screening**

| Items | Category | Females  n [%] | Males  n [%] | X^2^ | P-value |
| --- | --- | --- | --- | --- | --- |
| ***Help seeking behaviour*** | | | | | |
| If you had a symptom coming from the cervix or mouth of womb, would you ignore it? | Yes  No  Don’t know | 433 [61.3]  227 [32.2]  46 [6.5] | 575 [84.1]  55 [8.0]  54 [7.9] | 130.12 | **< 0.001** |
| If you had a symptom coming from the cervix or mouth of womb, would you try self-medication, for example get some ointment to apply from the local supermarket? | Yes  No  Don’t know | 95 [26.3]  233 [64.5]  33 [9.1] | 169 [69.3]  28 [11.5]  47 [19.3] | 176.59 | **< 0.001** |
| If you had a symptom coming from the cervix or mouth of womb, would you tell someone close to you? | Yes  No  Don’t know | 547 [78.6]  106 [15.2]  43 [6.2] | 523 [79.0]  77 [11.6]  62 [9.4] | 7.95 | **0.02** |
| If you had a symptom coming from the cervix or mouth of womb, would you visit a traditional healer? | Yes  No  Don’t know | 71 [10.3]  574 [83.6]  42 [6.1] | 55[8.5]  524 [81.4]  65 [10.1] | 7.93 | **0.02** |
| If you had a symptom coming from the cervix or womb, how soon would you visit a traditional healer?"  N = 135 [Said yes in No35] | < 1 week  ≥ 1 week < 1 month  ≥ 1 month  > 3 months | 37 [64,9]  15 [26.3]  3 [5.3]  2 [3.5] | 47 [88.7%]  4 [7.5%]  2 [3.8%] | 11.52 | **0.003** |
| If you had a symptom coming from the cervix or womb, how soon would you visit the pharmacy/clinic/health centre/hospital? | < 1 week  ≥ 1 week < 1 month  ≥ 1 month < 3 months  ≥ 3 months  Never | 247 [ 75.2]  54 [16.3]  12 [3.6]  4 [1.2]  13 [3.6] | 238 [ 75.8]  58 [18.5]  12 [3.8]  3 [1]  0 | 15.14 | 0.01 |
| ***Confidence and skills in relation to a sign/symptom of cervical cancer*** | | | | | |
| Are you confident that you would notice a symptom that could be cervical cancer? | Yes  No  Don’t know | 434 [62.0]  202 [28.9]  64[9.1] | 537 [78.7]  94 [13.8]  51 [7.5] | 50.06 | <0.001 |
| Have you ever been to see a nurse or clinical officer or doctor about a symptom that made you think something was wrong, like a symptom of cervical cancer? | Yes  No  Not noticed any symptoms or signs | 242 [34.2]  381 [54.0]  83 [11.8] | 271 [39.5]  324 [47.2]  91 [13.3] | 6.68 | 0.04 |
| Have you ever been to see a traditional healer about a symptom that made you think something was wrong, like a symptom of cervical cancer? | Yes  No  Not noticed any symptoms or signs | 85 [12.0]  534 [75.6]  87 [12.4] | 107 [15.7]  482 [70.9]  91 [13.4] | 3.96 | 0.14 |
| ***Barriers to seeking help*** | | | | | |
| “I would find it difficult to go for medical help because I would be worried about wasting the nurse/clinical officer/doctor’s time” | Agree  Disagree  Don't know | 233 [32.5]  446 [62.1]  39 [5.4] | 242 [35.3]  408 [59.5]  36 [5.2] | 1.57 | 0.46 |
| “I would find it difficult to go for medical help because I would be worried about what the nurse/clinical officer/doctor might find wrong” | Agree  Disagree  Don't know | 248 [34.5]  434 [60.4]  36 [5.0] | 237 [34.6%]  402 [58.8%]  45 [6.6%] | 1.58 | 0.46 |
| “I would find it difficult to go for medical help because I would be worried about what tests the nurse/clinical officer/doctor might do” | Agree  Disagree  Don't know | 250 [ 34.8]  423 [ 58.8]  46 [6.4] | 237 [34.5]  411 [ 59.8]  39 [5.7] | 0.06 | 0.97 |
| “I would find it difficult to go for medical help because I am too busy or have other things to worry about” | Agree  Disagree  Don't know | 235 [32.7]  446 [62.0]  38 [5.3] | 231 [33.6]  420 [61.0]  37[5.4] | 0.33 | 0.85 |
| “I would find it difficult to go for medical help because it takes too long to be seen at the clinic/health centre” | Agree  Disagree  Don't know | 255 [35.4]  432 [ 60.0]  33 [4.6] | 256 [37.3]  393 [57.3]  37 [5.4] | 1.43 | 0.49 |
| “I would find it difficult to go for medical help because we have no money for transport or the clinic/health centre charges” | Agree  Disagree  Don't know | 302 [42.0]  385 [53.5]  32 [4.5] | 265 [38.6]  384 [55.9]  38 [5.5] | 2.32 | 0.31 |
| “I would find it difficult to go for medical help because I would not feel confident talking about my symptoms” | Agree  Disagree  Don't know | 242 [33.6]  442 [61.3]  37 [5.1] | 242 [35.4]  403 [59.0]  38 [5.6] | 0.97 | 0.62 |
| “I would find it difficult to go for medical help because I have had a bad experience in the clinic/health centre in the past” | Agree  Disagree  Don't know | 255 [35.5]  429 [ 59.7]  35 [4.8] | 241 [35.2]  399 [58.2]  45 [6.6] | 2.15 | 0.34 |
| “I would find it difficult to go for medical help because I would feel embarrassed” | Agree  Disagree  Don't know | 233 [32.3]  450 [62.3]  39 [5.4] | 232 [34%]  406 [59.4%]  45 [6.6%] | 2.36 | 0.31 |
| “I would find it difficult to go for medical help because the nurse/clinical officer/doctor would not understand my language or culture” | Agree  Disagree  Don't know | 228 [31.8]  454 [ 63.2]  36 [5.0] | 227 [33.1]  420 [61.2]  39 [5.7] | 0.88 | 0.65 |
| “I would find it difficult to go for medical help because my husband/partner or family member would not allow me to go” | Agree  Disagree  Don't know | 240 [33.3]  453 [62.8]  28 [3.9] | 212 [30.9]  432 [62.9]  43 [6.3] | 3.96 | 0.14 |
| “I would find it difficult to go for medical help because I think that if I have a disease like cancer there is no use for the nurse/clinical officer/doctor and I will die anyway” | Agree  Disagree  Don't know | 227 [31.7]  462 [64.5]  27 [3.8] | 212 [30.9]  434 [63.3]  40 [5.8] | 3.70 | 0.16 |
